# Leadership for healthcare

**Published:** 2011-09-20

**Authors:** Amy Tan Bee Choo, Jason Cheah

**Affiliations:** Agency for Integrated Care, Republic of Singapore; Chief Executive Officer, Agency for Integrated Care, Republic of Singapore

*Leadership for Healthcare* offers a comprehensive summary of generic concepts and ideas about leadership and attempts to organise and structure the content based on the ‘Warwick 6 C Framework for thinking about leadership’ which was co-developed by the authors.

The book builds on the idea that too many earlier reviews of leadership approaches centred on separate individualistic models. Instead, it examines the evidence for a comprehensive leadership approach in the wider arena that healthcare leaders have to operate in.

This idea is not new, but the authors do provide an easy to use, robust and accessible review of the evidence. The ‘6-C Framework’ views leadership—chapter by chapter of the book—from six different angles: concepts, characteristics, contexts, challenges, capabilities and consequences and emphasises that these elements are inter-connected and inter-active.

- Conceptual approaches to leadership are personal qualities of the leader, organisational positions of a leader and leadership as a social process. The authors refer to several studies to clarify the conceptualisation of leadership being used in any given setting as an important pre-requisite for effective leadership development. We agree with the authors that if the concept of leadership is understood in the social processes of influence and mobilisation, then the attention will need to be paid to how the leader understands, interacts with and engages with the group being led. Leadership through influence requires the cultivation of interpersonal skills and emotional intelligence, amongst other aspects.
- Characteristics of leadership are categorised into formal and informal leadership, direct and indirect leadership, clinical and non-clinical leadership, individual and shared/distributed leadership, different bases of power. It was noted by the authors that the leadership characteristics may vary according to the role. Leadership development activities need to be geared to the roles and resources of those in leadership positions. While the authors show the conceptual distinction between leader development and leadership development is a useful one, both types of development are important, according to the context and the needs of the organisation. We agree that overall, leadership development requires careful thinking about who is to be developed, and what their potential roles and contributions are within and for the organisation. Different types of leaders are different sources and processes of influence, and it is necessary for leadership development to be designed appropriately.
- Context of leadership shows the importance of partnership and inter-professional and inter-organisational networks as critical skills for healthcare leaders, particularly but not exclusively at senior levels. We like the way the authors highlight the unique importance of partnership as a crucial context to the leadership in healthcare, the need to focus on whole-system design and development, to ensure that partnership contributes to strategic purpose.
- Challenges of leadership show three key challenges: sense-making and constitutional challenge leading networked and partnership organisations, challenge of turn-around and leading organisations out of failure and the challenge of leading change, innovation and improvement. These are particularly relevant to leaders who work with and within networks of providers to foster better integrated care for patients. While this aspect is perhaps the most crucial and relevant to a leader working within an integrated delivery system, the chapter is not sufficiently detailed in terms of content for useful and practical lessons to be learnt here.
- Capabilities of leadership subsume traits, behaviours (competencies, emotional intelligence, leadership with political awareness, overarching competencies), capability of leading networks and teams, transformational and transactional leadership behaviours and styles and post-transformational leadership. These concepts are not new and are perhaps the most widely written on in the popular management literature and academic journals.
- Consequences of leadership describes how leadership has an impact on people and systems, talks about causes and effects, inputs, activities and outputs, customer satisfaction and eventually outcomes in a so-called public value stream. This chapter emphasises the impact of leadership on organisational performance and health/system outcomes.

There is also a chapter on leadership development towards the end of the book which attempts to offer an outline of leadership development alternatives for the ‘Warwick 6 C Framework’ in a step-by-step manner. For each of the above mentioned framework elements, leadership development options are discussed. The authors emphasise that clear thinking about leadership development is essential. The book provides a simple analytical framework to ask critical questions to ensure alignment between strategic purposes and leadership development practices.

This book undertakes a huge task, i.e. offering a comprehensive framework to structure the more or less recent and important thoughts, write-ups and research results on leadership. The best part of the book is, however, the final chapter when the authors attempt to draw practical conclusions out of the very theoretical ‘6 C Framework’.

Hence, this book is suitable for someone who is perhaps more interested in research in theoretical and very abstract disquisitions. It is not recommended for the practising leader in the healthcare sector who wants to understand his knowledge, skill and performance gaps and who strives to enhance his own capabilities. The practical value of this book for a leader working in healthcare is hence quite limited. Only the final chapter of the book can be suggested as reading material for those who are in charge of structuring leadership development programs for healthcare sector—if they have some time to spare. In this case, you would find recommendations such as:

- Planning for leadership development needs to cover: how people are selected; the curriculum design; the pedagogical principles; the actual activities; the organisational framework; and how leadership development is evaluated.
- Clarify the concept of leadership—otherwise the appropriate may be inappropriate for the needs of the organisation. How clear is the organisation about its views of what constitutes effective leadership and what constitutes effective management.
- Cross-sector leadership development may be particularly important to help develop skills in emotional intelligence and leadership with political awareness.
- Designing in evaluation at an early stage will help ensure that leadership development is focused and that it can be modified over time using systematic feedback.

In general, the authors list a multitude of different facets of leadership and evidence that support this or that model, hypothesis or statement without adding much of their own opinion. At the end the reader has the impression that everything has been considered but not too much has really been suggested, recommended or concluded.

We would rate this book 3 stars out of 5.

